# Compassion toward others and self-compassion predict mental and physical well-being: a 5-year longitudinal study of 1090 community-dwelling adults across the lifespan

**DOI:** 10.1038/s41398-021-01491-8

**Published:** 2021-07-13

**Authors:** Ellen E. Lee, Tushara Govind, Marina Ramsey, Tsung Chin Wu, Rebecca Daly, Jinyuan Liu, Xin M. Tu, Martin P. Paulus, Michael L. Thomas, Dilip V. Jeste

**Affiliations:** 1 Department of Psychiatry, University of California San Diego, La Jolla, CA USA; 2 VA San Diego Healthcare System, San Diego, CA USA; 3 Sam and Rose Stein Institute for Research on Aging, University of California San Diego, La Jolla, CA USA; 4 Department of Family Medicine and Public Health, University of California San Diego, La Jolla, CA USA; 5 Laureate Institute for Brain Research, Tulsa, OK USA; 6 Department of Psychology, Colorado State University, Fort Collins, CO USA; 7 Department of Neurosciences, University of California San Diego, La Jolla, CA USA

**Keywords:** Predictive markers, Prognostic markers

## Abstract

There is growing interest in the role of compassion in promoting health and well-being, with cross-sectional data showing an inverse correlation with loneliness. This is the first longitudinal study examining both compassion toward others (CTO) and compassion toward self (CTS) as predictors of mental and physical health outcomes including loneliness, across adult lifespan. We followed 552 women and 538 men in San Diego County for up to 7.5 (mean 4.8 and SD 2.2) years, using validated rating scales for CTO, CTS, and loneliness. Linear mixed-effects models were employed to examine age- and sex-related trajectories of CTO and CTS over time. Linear regression models were used to evaluate baseline and longitudinal relationships of CTO and CTS with mental well-being, physical well-being, and loneliness. CTS and CTO were weakly intercorrelated. Women had higher baseline CTO than men. While CTO was stable over time and across the lifespan, CTS scores had an inverse U-shaped relationship with age, peaking around age 77. There were significant baseline × slope interactions of both CTO and CTS predicting improvements in physical well-being in adults <60 years old. Increases in CTO and CTS predicted improvements in mental well-being. Higher baseline CTO and CTS as well as increases in CTO and CTS scores predicted lower loneliness scores at follow-up. Thus, CTO and CTS were associated with better mental well-being and loneliness across the adult lifespan, and physical well-being in younger adults, and are promising targets for interventions to improve health outcomes.

## Introduction

Pro-social attitudes and behaviors like compassion have been linked to greater well-being and better health in individuals and potentially, in the society [1, 2]. Compassion involves recognizing suffering of others and then taking action to help them [3]. A related construct is empathy, which enables individuals both cognitively and emotionally to identify and experience the mental states of other people, allowing comprehension of and engagement in social relationships [4]. Empathy is necessary for compassion but is not sufficient because the latter also requires motivation followed by action. Compassion is considered to include two subtypes, according to the target of compassion—compassion toward others (CTO) and compassion toward self (CTS) [3, 5].

From an evolutionary perspective, CTO has been purported to support caring for vulnerable offspring (as human infants and children require a much higher level and longer duration of care relative to other mammals), aid mate selection, and increased cooperation between non-related individuals [5]. CTS may also have a beneficial role of tempering self-criticism. Self-criticism can be important for stimulating threat processing although it can also be associated with increased anxiety and depression [6].

Compared to men, women have been reported to have greater levels of affective and cognitive empathy as well as higher CTO [7–10], but lower CTS [11–17]. From an evolutionary standpoint, only women have childbearing roles, and traditionally they have also had greater caregiving responsibilities. Therefore, higher CTO levels may be associated with female sex both biologically and culturally. The reported relationships of CTO and CTS with age has been mixed, though longitudinal studies are lacking. While cross-sectional studies of CTO [3, 9, 10] and CTS [16, 18, 19] have reported similar levels among older versus younger adults, several cross-sectional studies of empathy have demonstrated lower levels of cognitive empathy [20–25], but similar levels of affective empathy in older adults [4, 22, 25–28]. The few published longitudinal studies of empathy in older adults have had mixed findings: five with increasing empathy with aging (especially after age 40) [29], one with decline in empathy (especially in women) [30], and one with stable levels of empathy with aging [31]. These longitudinal findings are limited by somewhat varied definitions of empathy, use of different measures, unbalanced sex distributions [29, 30], insufficient racial/ethnic/socioeconomic diversity in the sample, and a lack of meaningful health outcomes. Similarly, the “Grandmother Hypothesis” [32] posits that older women of post-reproductive ages support the species through helping raise grandchildren, enhancing fertility of their adult daughters, and survival and longevity of the grandchildren [33]. Thus, from an evolutionary standpoint, CTO and CTS may have important roles across the lifespan and in both sexes, though prior studies have not examined sex- and age-specific changes in CTO and CTS.

During recent decades there has been a behavioral pandemic of loneliness, associated with higher morbidity and all-cause mortality [34, 35]. Loneliness is defined as subjective distress arising from discrepancies between desired and perceived social relationships [36], and has somewhat distinct health consequences from social isolation which refers to an objective lack of social contact and support [34]. The increased mortality from loneliness and associated suicides and opioid-related deaths has led to lowering the average US lifespan two years in a row after a progressive increase since 1959 [37]. Our earlier cross-sectional study suggested three age groups in which loneliness seemed to peak—late 20s, mid-50s, and late 80s [35]. A consistent finding in four published studies has been a strong inverse correlation between loneliness and wisdom, a multi-component personality trait, and its main component—pro-social behaviors of empathy and compassion [35, 38–40]. A recent study using EEG also provided neurobiological support to the negative association between these traits [41]. Another study found that loneliness and wisdom, especially its compassion component, were associated with lower versus higher, respectively, levels of alpha and beta diversity of gut microbial taxa composition [42].

Cross-sectional studies have reported associations of compassion with better mental health (greater happiness and well-being [43–45]) and physical health (lower cardiovascular risk [2] and decreased inflammation [46]). However, to our knowledge, the interrelationship of CTO and CTS, how CTO and CTS change across the adult lifespan, as well as their possible association with mental (including loneliness) and physical health have not been examined longitudinally in sizable cohorts of women and men. Pro-social attitudes and behaviors are partially modifiable [47–54] and should be an excellent potential target for health-focused interventions across the adult lifespan. Interventions to enhance CTO and CTS have been shown to improve health outcomes [47–54]. For example, two studies reported that a Mindfulness Self-Compassion Program improved CTS and also reduced diabetes distress and hemoglobin A1C in adults with diabetes [47, 55]. While promising, the published interventions have usually not examined the associations with age and sex, had relatively short follow-up periods of only a few months, and did not assess both CTO and CTS using validated measures. The longer-term sex- and age-related associations between CTO, CTS, and health are unknown. It is not clear what are the relative importance of the relationship of CTO and CTS to physical and mental health, including loneliness.

In the current study, CTO and CTS were assessed in a relatively large sex-balanced community-based sample across the adult lifespan (age range 27–101 years) that was followed longitudinally for up to 7.5 (mean 4.8 and SD 2.2) years. We sought to examine: (1) the relationship between CTO and CTS; (2) sex differences in CTO and CTS; (3) how CTO and CTS vary by sex across the lifespan; and (4) the relationship of CTO and CTS to physical and mental health including loneliness. We hypothesized that (1) baseline CTO and CTS levels would be highly intercorrelated, and (2) women would have higher levels of CTO than men. We explored (3) how CTO and CTS would change longitudinally by sex and across the age-span. Finally, we hypothesized that (4) higher baseline levels and longitudinal increases in CTO and CTS would predict higher levels of mental well-being including lower loneliness, as well as better physical health at the end of the follow-up period, controlling for smoking, alcohol use, and relevant sociodemographic factors.

## Methods

### Study participants

The study participants were recruited from the UCSD Successful AGing Evaluation (SAGE) study across the adult lifespan, which has been described previously [56, 57]. Briefly, participants were recruited using list-assisted random digit dialing in San Diego county. The inclusion criteria were (1) community-dwelling adults, (2) aged 21–100+ years, (3) provision of written informed consent to participate in the study, (4) fluency in English, (5) a telephone line at home, (6) physical and mental abilities to complete the study assessments, and (7) no known diagnosis of dementia. Persons who lived in nursing homes or required daily skilled nursing care, or had a terminal illness, were excluded. All participants included in the current study had CTO and CTS assessments and annual follow-up data available (two or more data-points).

The study protocol was approved by the UC San Diego Human Research Protections Program (HRPP) and all the participants provided a written informed consent prior to study participation. The data were collected over a period from July 2012 through February 2020.

### Sociodemographic and clinical characteristics

Trained study staff completed a 25-min initial phone interview followed by a survey that was mailed or completed online. Data included sociodemographic information on age, sex, education level, race/ethnicity, current marital status, living situation, and income. Self-administered standardized assessments were completed for depression (Patient Health Questionnaire-9 or PHQ-9) [58], anxiety (Brief Symptom Inventory – Anxiety subscale) [59], perceived stress (Perceived Stress Scale) [60], resilience (Connor Davidson Resilience Scale or CD-RISC) [61], optimism (Life Orientation Test – Revised or LOTR) [62], and satisfaction with life (Satisfaction with Life Scale or SWLS) [63]. Loneliness was measured only at the end of the study, with the 20-item UCLA-3 Loneliness Scale [64]. Physical and mental health assessments included mental and physical well-being based on the composite scores from the Medical Outcomes Survey—Short Form 36 (SF-36) [65].

CTO was assessed with the Santa Clara Brief Compassion Scale, a 5-item scale with items rated with 7-point Likert scale (1: “Not at all true of me” to 7: “Very true of me”) [8]. This validated measure was based on the 21-item Sprecher and Fehr Compassionate Love for Humanity Scale [7], and is highly correlated with the original version (*r* = 0.96) with high internal reliability (Cronbach’s alpha of 0.90). An example item is “I tend to feel compassion for people, even though I do not know them.” To measure CTS, we used the Neff Self-compassion Scale short-form, a 12-item adaptation of the popular and well-validated 25-item scale [66], that has strong correlation to the original version (*r* ≥ 0.97) and strong internal consistency (Cronbach’s alpha ≥0.86). The items are rated on a 5-point Likert scale (1: “Almost never” to 5: “Almost always”). An illustrative item is “When I’m going through a very hard time, I give myself the caring and tenderness I need.”

The participants were followed longitudinally for up to 7 years with assessments being performed at Years 0 (baseline), 1, 2, 3, 6, and 7.

### Statistical analyses

Independent sample *t*-tests and chi-square tests were used to assess differences in sociodemographic factors and various outcome measures between male and female participants. Log transformation was used for non-normally distributed variables. To address Question 1, the baseline relationship between CTO and CTS was assessed using Spearman’s correlation. For Question 2, we used independent sample *t*-tests to compare CTO and CTS by sex. For Question 3, linear mixed-effects models were used to determine trajectories of CTO and CTS over time (polynomial), with age, sex, marital status, race, and household income at baseline as the predictors, along with interactions of time with each of the demographic variables. Two random-effects structures were chosen with one including a random intercept only and the other including both a random intercept and random slope. The optimal structure was selected by using REML AIC [67]. Sandwich variance estimates were used to improve inference validity.

For Question 4, multiple linear regression models were developed to examine associations of the individual baseline and slope values of CTO and CTS calculated based on the baseline and last observed outcomes model with changes in mental and physical health. Inference was based on estimating equations to improve inference validity [68]. To account for moderation effect by baseline CTO and CTS, the model also included baseline by slope interaction for CTO and CTS. All models controlled for age, sex, marital status, race, and household income at baseline [69]. Due to a previously reported beginning of a sharp decline in physical well-being around age 60 in our sample [63], longitudinal models of physical well-being were run separately for individuals ≤60 years old and those >60 years. The regression model was also used to assess associations of CTO and CTS with loneliness at follow-up. The analyses were repeated by adding smoking and alcohol use at baseline as additional covariates.

We present effect sizes and *p*-values for all of these statistical tests, and interpret small-medium effect sizes (i.e., Cohen’s *d* > 0.20 or *r* ≥ 0.30) as meaningful. Statistical significance was defined as alpha = 0.05 (two-tailed) for all analyses.

## Results

### Sample characteristics

Our sample consisted of comparable numbers of women (*N* = 538) and men (*N* = 552). The men were significantly older, had more education and higher household income, and were less likely to be non-Caucasian or in a marriage-like relationship (Table 1). Women had significantly higher anxiety, but depression levels were similar between the sexes.

**Table 1:** Baseline demographic and clinical comparison of women and men in the study sample.

	Women			Men						
	*N*	Mean or %	SD	*N*	Mean or %	SD	*t* or *X*^2^	df	*p*	Cohen’s *d*
*Sociodemographic*										
Age at baseline visit (years)	552	61.5	22.0	538	66.0	20.3	−3.51	1088	<0.001	−0.21
Race (% Caucasian)	403	73.1		421	78.5		4.33	1	0.04	
Education (%)							20.0	1	<0.001	
High School & below	79	14.5		56	10.5					
Some college to bachelor degree	367	67.3		321	60.1					
Post-graduate degree	99	18.2		157	29.4					
Household income (%)							14.2	2	0.001	
<$35,000	143	28.9		100	19.8					
$35,000–$74,999	161	32.5		160	31.7					
$75,000+	191	38.6		245	48.5					
Marital status (% marriage-like relationship)	255	46.6		384	71.5		70.9	1	<0.001	
Currently employed (% no)	411	75.0		377	70.6		2.65	1	0.10	
Smoking (% ever)	189	34.2		264	49.1		24.7	1	<0.001	
Alcohol use							20.0	3	<0.001	
Lifetime abstainers	84	15.9		66	12.6					
Infrequent drinkers	290	55.0		241	45.9					
Regular drinkers	90	17.1		144	27.4					
Former drinkers	63	12.0		74	14.1					
*Psychopathology*										
Anxiety (BSIA)	541	2.1	3.3	529	1.5	2.4	2.98	1068	0.003	0.18
Depression (CESD)	529	5.4	4.6	526	5.0	4.4	1.65	1053	0.10	0.10
*Compassion measures*										
CTS (Neff Scale)	531	41.4	8.0	515	42.5	7.2	−2.22	1044	0.03	−0.14
CTO (SCBCS)	539	5.0	1.2	527	4.4	1.4	6.93	1064	<.001	0.42
*Outcomes*										
Physical well-being (SF-36)	533	46.8	11.1	520	47.1	10.6	−0.35	1051	0.73	−0.02
Mental well-being (SF-36)	533	52.5	9.5	520	54.2	7.3	−3.19	1051	0.001	−0.20
Loneliness (UCLA)	318	36.4	10.5	319	36.7	10.3	−0.36	635	0.72	−0.03

*BSIA* Brief Symptom Inventory – Anxiety subscale, C*ESD* Center for Epidemiologic Studies Depression Scale, *CTO* compassion toward others, *CTS* Compassion toward self, *SCBCS* Santa Clara Brief Compassion Scale, *SF-36* 36-item Short Form Survey, *UCLA* UCLA Loneliness Scale [assessed only at follow-up].

### Question 1: Intercorrelation of CTO and CTS at baseline

CTO and CTS were weakly correlated to each other (*r* = 0.16, *p* ≤ 0.001 in the whole sample; *r* = 0.09, *p* = 0.02 in women; *r* = 0.20, *p* < 0.001 in men). CTS and CTO were more strongly intercorrelated within men (*z* = −1.99, *p* = 0.02).

### Question 2: Baseline sex differences in CTO and CTS

Baseline CTS scores did not differ significantly by sex; however, women had significantly higher CTO than men (5.0 ± 1.2 vs. 4.4 ± 1.4, *t*_1,064_ = 6.93, *p* < 0.001, *d* = 0.42).

### Question 3: Longitudinal relationships of compassion with sex and age

**Fig. 1:**
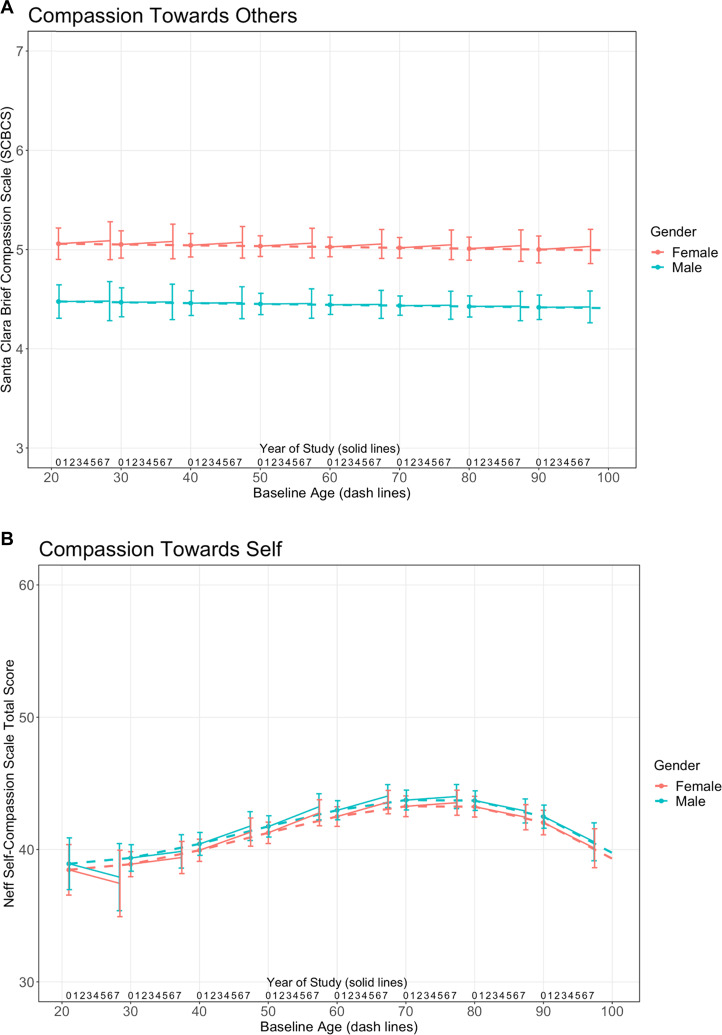
Trajectories of compassion toward others (CTO) and compassion toward self (CTS) by age and gender. **A** Trajectories of compassion toward others (CTO) by Age and Gender (Mean follow-up 4.8 years, up to 7 years). Solid lines depict the changes in CTO levels up to 7 years of follow-up by decade of age. Dotted lines show the best-fit model of CTO with baseline age. **B** Trajectories of compassion toward self (CTS) by age and gender (mean follow-up 4.8 years, up to 7 years). Solid lines depict the changes in CTS levels up to 7 years of follow-up by decade of age. Dotted lines show the best-fit model of CTS with baseline age.

Figure 1 shows changes in CTO and CTS plotted against follow-up year and against baseline age, based on results from the linear mixed-effects model. Women had significantly higher CTO than men throughout the follow-up period and across all age groups (*B* = −0.56, SE = 0.07, *t*_1,065_ = −7.86, *p* ≤ 0.001) (Fig. 1A). Follow-up year had a non-linear association with CTO (*B* = 0.006, SE = 0.001, *t*_3,156_ = 5.06, *p* ≤ 0.001), though 7-year changes in CTO were modest across age groups. Non-Caucasian individuals had higher CTO levels than Caucasians, and lifetime abstainers had higher CTO levels than current regular drinkers. Baseline age, marital status, income, smoking, and infrequent/former drinking were not related to changes in CTO.

Sex and follow-up year were not significantly related to CTS levels (*B* = 0.28, SE = 0.42, *t*_1,041_ = 0.67, *p* = 0.50; and *B* = 0.02, SE = 0.04, *t*_3,029_ = 0.48, *p* = 0.63; respectively). Baseline age had a cubic association with CTS (*B* = −0.0007, SE = < 0.001, *t*_1,041_ = −2.87, *p* = 0.004), with peak CTS at age 77. At baseline age in the 40s and 60s, CTS increased slightly throughout the follow-up period, while it remained stable among participants in their 20s and 90s (Fig. 1B). Annual household income >$35,000 was associated with higher CTS. Marital status, race, smoking, and alcohol use were not related to changes in CTS.

### Question 4a: Longitudinal associations of compassion with physical and mental well-being

The models of physical well-being differed by age group. In the younger age group (≤60 years old), baseline values as well as changes in CTO and CTS were associated with changes in physical well-being (Table 2A). There was a significant interaction between baseline CTO and slope of CTO (*B* = −0.74, SE = 0.25, *η*_p_^2^ = 0.025), such that individuals with higher baseline CTO had less robust CTO-related increases in physical well-being. Conversely, individuals with lower baseline CTO had more robust CTO-related increases in physical well-being. There was also a significant baseline CTS × slope of CTS interaction (*B* = 0.03, SE = 0.009, *η*_p_^2^ = 0.041), such that individuals with lower baseline CTS had less robust CTS-related increases in physical well-being. The effect sizes for the CTO and CTS variables in predicting physical well-being were greater than those of smoking and drinking alcohol. The model that included CTO and CTS had a better model-fit compared to the model without CTO and CTS [goodness-of-fit metrics (QIC) were 1919 and 2126, respectively].

**Table 2:** Multiple linear regression models of compassion toward others (CTO) and compassion toward self (CTS) with the outcomes of physical well-being among individuals age ≤60 years (A) outcomes of physical well-being among individuals age >60 years (B), and mental well-being (C).

	Prediction of change in physical well-being over 5 years				
	*B*	SE	Wald	*p*	*η*_p_^2^
(A) Baseline and changes in compassion toward others (CTO) and compassion toward self (CTS) as predictors of change in physical well-being over 7-year follow-up in individuals age ≤60 years					
Intercept	0.44	0.91	0.23	0.63	
Baseline CTO	0.13	0.09	1.91	0.17	0.002
Slope CTO	3.19	1.02	9.77	**0.002**	<0.001
Baseline CTS	−0.03	0.02	2.29	0.13	0.006
Slope CTS	−1.25	0.38	10.71	**0.001**	0.05
Age	−0.01	0.01	1.25	0.26	0.004
Gender (men)^a^	0.28	0.25	1.32	0.25	0.004
Marital status (marriage-like)^b^	−0.01	0.30	<0.01	0.96	<0.001
Race (Caucasian)^c^	0.05	0.26	0.03	0.86	<0.001
Household income^d^					0.01
$35,000–$74,999	−0.56	0.47	1.39	0.24	
≥$75,000	0.02	0.36	<0.01	0.95	
Smoking (ever)^e^	0.002	0.31	<0.01	0.99	<0.001
Alcohol use^f^					0.006
Infrequent drinkers	0.36	0.30	1.40	0.24	
Regular drinkers	0.09	0.40	0.05	0.83	
Former drinkers	0.62	0.52	1.39	0.24	
Baseline CTO*Slope CTO	−0.74	0.25	8.99	**0.003**	0.025
Baseline CTS*Slope CTS	0.03	0.009	8.13	**0.004**	0.041
(B) Baseline and changes in compassion toward others (CTO) and compassion toward self (CTS) as predictors of change in PHysical Well-being over 7-year follow-up in individuals age >60 years					
Intercept	−1.23	2.05	0.36	0.55	
Baseline CTO	−0.25	0.15	2.99	0.08	0.007
Slope CTO	0.79	0.94	0.71	0.40	0.006
Baseline CTS	−0.02	0.03	0.36	0.55	0.001
Slope CTS	0.31	0.26	1.43	0.23	0.031
Age	0.03	0.02	1.43	0.23	0.004
Gender (men)^a^	−0.32	0.36	0.83	0.36	0.002
Marital status (marriage-like)^b^	0.66	0.43	2.37	0.12	0.006
Race (Caucasian)^c^	0.26	0.38	0.48	0.48	0.001
Household income^d^					0.011
$35,000–$74,999	−0.77	0.54	2.03	0.15	
≥$75,000	−0.01	0.55	<0.01	0.98	
Smoking (ever)^e^	0.41	0.35	1.38	0.24	0.003
Alcohol use^f^					0.001
Infrequent drinkers	0.10	0.53	0.03	0.86	
Regular drinkers	0.07	0.55	0.01	0.90	
Former drinkers	−0.13	0.73	0.03	0.86	
(C) Baseline and changes in compassion toward others (CTO) and compassion toward self (CTS) as predictors of change in mental well-being over 7 years					
Intercept	−0.05	0.12	0.18	0.68	
Baseline CTO	0.01	0.01	0.98	0.32	0.002
Slope CTO	0.13	0.03	4.37	**0.04**	0.02
Baseline CTS	−0.001	0.002	<0.01	0.95	<0.001
Slope CTS	0.05	0.02	6.46	**0.01**	0.098
Age	−0.001	0.001	1.92	0.17	0.004
Gender (men)^a^	0.02	0.03	0.24	0.63	0.001
Marital status (marriage-like)^b^	−0.01	0.04	0.11	0.74	<0.001
Race (Caucasian)^c^	0.03	0.03	1.08	0.30	0.001
Household income^d^					0.003
$35,000–$74,999	−0.001	0.04	<0.01	0.98	
≥$75,000	0.04	0.04	0.78	0.38	

*CTO* compassion toward others, *CTS* compassion toward self.

^a^Compared to women, ^b^compared to single, ^c^compared to non-Caucasian, ^d^compared to <$35,000, ^e^compared to never, ^f^compared to lifetime abstainers.

Bold values indicates statistically significant *p* values <0.05.

In the older group (>60 years old), baseline and changes in CTS and CTO were not significantly associated with changes in physical well-being (Table 2B).

Greater increases in CTO and CTS predicted improvements in mental well-being (Table 2C). There was no significant interaction between baseline scores and subsequent changes in CTO or CTS scores.

### Question 4b: Longitudinal association of compassion with loneliness

Higher baseline CTO and CTS, as well as greater increases in CTO and CTS scores significantly predicted lower loneliness scores at the last follow-up (Table 3, with small effect sizes for CTO and medium effect sizes for CTS). Being male, being single at baseline, and having annual household incomes <$75,000 were associated with higher loneliness scores at the last follow-up (with small effect sizes). Infrequent drinkers, regular drinkers, and former drinkers all had significantly lower loneliness scores than lifetime abstainers. Race, age, and smoking habits did not have a significant relationship to loneliness.

**Table 3:** Best-fit linear model of compassion toward others (CTO) and compassion toward self (CTS) with loneliness at follow-up.

	Loneliness at follow-up				
	*B*	SE	Wald	*p*	*η*_p_^2^
Intercept	76.13	3.12	594.81	**<0.001**	
Baseline CTO	−0.76	0.31	6.19	**0.01**	0.01
Slope CTO	−5.36	2.26	5.62	**0.02**	0.01
Baseline CTS	−0.66	0.05	152.53	**<0.001**	0.22
Slope CTS	−3.34	0.44	56.49	**<0.001**	0.13
Age	−0.04	0.02	2.60	0.11	0.005
Gender (men)^a^	2.01	0.76	6.92	**0.009**	0.01
Marital status (marriage-like)^b^	−3.47	0.84	17.09	**<0.001**	0.03
Race (Caucasian^c^	0.14	0.86	0.02	0.87	<0.001
Household income^d^					0.01
$35,000–$74,999	−1.38	1.14	1.46	0.23	
≥$75,000	−2.64	1.18	4.97	**0.03**	
Smoking (ever)^e^	−0.59	0.76	0.61	0.44	0.001
Alcohol use^f^					0.01
Infrequent drinkers	−2.78	1.13	6.08	**0.01**	
Regular drinkers	−3.54	1.27	7.75	**0.005**	
Former drinkers	−3.29	1.47	4.99	**0.03**	

*CTO* compassion toward others, *CTS* compassion toward self.

^a^Compared to men, ^b^compared to single, ^c^compared to non-Caucasian, ^d^compared to <$35,000, ^e^compared to never, ^f^compared to lifetime abstainer.

Bold values indicates statistically significant *p* values <0.05.

## Discussion

The current study aimed to examine the baseline and longitudinal sex- and age-related associations of CTO and CTS with mental and physical health, and loneliness. Our findings show weak correlation between CTO and CTS, clear sex differences in baseline levels as well as longitudinal trajectories of CTO, and an inverse-U association of CTS with age, and significant associations of CTO and CTS with physical well-being in younger adults as well as with mental well-being and loneliness across the lifespan. Overall, these findings partially support our hypotheses. While CTO and CTS are not highly intercorrelated, CTO and CTS are closely and independently linked to health and loneliness, though sex and age appear to influence those associations.

While, to our knowledge, there have been no multi-year longitudinal follow-up studies of CTO and CTS across the adult lifespan, we did find a few published longitudinal studies examining changes in empathy with aging [29–31, 70–73]. Five longitudinal studies reported increases in empathy with aging [29, 70–73], while two found either no change or a small linear decline in empathy [30, 31]. The current findings of an inverted-U-shaped relationship between age and CTS are similar to those of O’Brien and colleagues from a sample of adults age 18–90 years, with middle-aged adults having greater affective and cognitive empathy than younger and older adults [74]. Grühn and colleagues found cross-sectional age differences but no changes longitudinally, and attributed their findings to cohort differences in empathy levels in a sample aged 15–87 years. Researchers have attributed changes in empathy to social networking use, media and technology consumption, and changes in other psychological characteristics and behaviors, parenting, and family practices, and the broader cultural zeitgeist (e.g., a culture of success or a culture of social consciousness) [29]. In this study, the decline in CTS after age 77 may reflect age-related declines in cognitive empathy and social cognition or generational differences [31, 74], though this study did not explicitly examine social cognition measures. For example, the middle-aged group in this sample had formative experiences, such as coming of age during the Civil Rights Movement, that may have influenced their development of CTS. Thus, both aging and cohort effects are important to consider for CTO and CTS.

Cross-sectional studies have reported higher empathy among women on both cognitive and affective empathy measures [20, 74–79]. Schieman and Van Gundy reported that the gap between the sexes closed in older ages [20]. Women have also been reported to have higher CTO than men across a number of cross-sectional studies in community-dwelling populations [7–10, 80], healthcare professionals [81], and younger adults [82]. as well as lower CTS than men in a meta-analysis [17]. Most of the longitudinal studies found higher empathy levels among women compared to men in their samples [29, 70–73] with one report of no sex difference across the lifespan [31]. Some of these longitudinal samples lacked sex balance, especially in older ages [29, 30], a wide range of socioeconomic status, and racial/ethnic diversity—though these appear to be key covariates of CTO and CTS.

While the current study found higher annual income (>$35,000) to be associated with higher CTS but not CTO, the literature linking CTO/CTS to education and socioeconomic status (SES) is complex and mixed. Cross-sectional studies reported that individuals with more education and higher SES had lower CTO [83] and higher CTS levels [84], which may be linked to the communal advantage of pro-social behaviors in communities with low SES [85]. Conversely, higher SES may reduce reliance on others and result in lower CTO [86], though the decreased parental stress and increased parental support in high SES environments during childhood may promote pro-social behaviors [87]. A 32-year longitudinal study reported that high childhood SES predicted higher CTO at age 30–40 and higher adulthood CTO was linked to higher adulthood SES at 10-year follow-up [87].

CTO and CTS represent distinct constructs. CTO has sometimes been associated with negative outcomes. For instance, one study found that caregivers with higher CTO experienced more intrusive thoughts about their care recipients’ condition than caregivers with lower CTO, regardless of the level of physical suffering that they saw in the care recipient. In concordance with the current findings, three observational studies of adults across the lifespan, college undergraduates, and nursing students reported weak or non-significant correlations between CTS and CTO [84, 88, 89]. However, compassion-based interventions may improve both CTO and CTS. A meta-analysis of such interventions (e.g., Compassion Focused Therapy, Mindful Self-Compassion) to enhance CTO or CTS found an overall improvement in both CTO and CTS, as well as overall well-being [90]. Our results highlight the sex differences in the CTO-CTS correlations, which could reflect baseline sex differences in CTO and CTS or sex-specific influences that affect CTO and CTS levels. These findings warrant further investigations in sex-related contributory factors.

The influence of CTO and CTS on physical health was consistent with prior studies which showed that CTS improved lifestyle behaviors and metabolic biomarkers among adults with diabetes [47, 55, 91]. Reviews have found that CTS interventions can improve health behaviors from goal-setting and monitoring [92] to affective responses [93]. These psychosocial determinants of health are important factors to consider for improving health and well-being across the lifespan. However, a case can also be made for reverse causality, i.e., individuals with better mental and physical health have greater CTO and CTS. The current study’s longitudinal findings appear to support a ceiling effect with CTO, such that individuals with higher CTO at baseline did not have CTO-related improvement in physical health. On the other hand, the longitudinal findings appear to have a floor effect with CTS, such that individuals with lower CTS at baseline did not have CTS-related improvement in physical health. Furthermore, CTO and CTS were not linked to physical health in adults 60+ years of age, likely due to the large number of confounding factors with older age. Randomized controlled trials (RCTs) of CTO and CTS interventions are warranted to specifically examine whether CTO and CTS are causally linked to health.

Our finding of both CTO and CTS being predictors of lower loneliness at follow-up provides support to the previous findings of a significant inverse correlation (*r* = 0.51–0.76) between loneliness and compassion or wisdom in at least four different cross-sectional studies [35, 38–40]. Though further empirical evidence is warranted, compassionate behavior can be a less socially threatening way to connect with others, as it is likely to be well-received and reciprocated [94]. Furthermore, CTO and CTS are reflective of empathic abilities (understanding the emotions and perspectives of others) that may bolster more rewarding social relationships. There is a possible neurobiological basis for a counteracting effect of compassion on loneliness. A recent EEG study found that measures of loneliness and wisdom (especially its compassion component) were related to contrasting modulations of cognitive processes (reduced versus enhanced response speed biased by angry versus happy emotions, respectively) and invoked similar (temporo-parietal junction) and distinct (superior parietal vs. insula, respectively) neural circuits in specific emotional contexts [47].

These findings support a need for broad investigations into the naturalistic development of CTO and CTS and how these traits may vary by cohort and environmental factors (using longitudinal studies in diverse cohorts from the community), biological mechanisms linked to developing CTO and CTS (inclusion of relevant biomarkers in longitudinal studies as well as RCTs of neurobiological compassion-focused interventions such as brain stimulation and biofeedback), and causal links between CTO/CTS and health (RCTs of compassion-based interventions with adequate follow-up periods).

This study has several limitations. The sample was predominantly Caucasian, and the results may not generalize to racial/ethnic minorities or to non-English-speaking adults. All the clinical assessments were self-report-based and did not include objective measures. We did not evaluate empathy in this study. Also, we did not begin assessing loneliness until the last follow-up; therefore, the contribution of CTO and CTS to the longitudinal changes in loneliness is not known. Also, we did not examine biomarkers of inflammation or other markers of stress or aging. The maximum longitudinal follow-up period was 7.5 years, and it is possible that longer follow-up might have produced different results.

Nonetheless, the study also has several strengths. To our knowledge, this is the first multi-year longitudinal study of CTO and CTS across the adult lifespan. It was based on a community-based sample selected using random digit dialing, with comparable numbers of women and men. We used validated rating scales and controlled for various sociodemographic factors.

Future research is warranted to identify the innate personality features and environmental influences (life events, relationships, cultural and societal norms) that may alter CTO and CTS in diverse populations. In that vein, the benefits of CTO and CTS interventions have not been fully characterized—i.e., whether these interventions have lasting effects on health and other outcomes and which individuals benefit most from such interventions. Last, using biomarkers and objective assessments of CTO and CTS can improve our understanding of the biological underpinnings of pro-social behaviors. Efforts to promote compassion at individual and societal levels may help stem the modern behavioral pandemics of loneliness, stress, suicides, and opioid abuse, worsened by the recent Covid-19 pandemic and the necessary social distancing requirements [95, 96].
